# Effectiveness of an expert assessment and individualised treatment compared with a minimal home-based exercise program in women with late-term shoulder impairments after primary breast cancer surgery: study protocol for a randomised controlled trial

**DOI:** 10.1186/s13063-022-06659-1

**Published:** 2022-08-20

**Authors:** Kim Michéle Feder, Hans Bjarke Rahr, Marianne Djernes Lautrup, Heidi Klakk Egebæk, Robin Christensen, Kim Gordon Ingwersen

**Affiliations:** 1 Department of Physiotherapy, Vejle Hospital, University Hospital of Southern Denmark, Beriderbakken 4, 7100 Vejle, Denmark; 2 Department of Regional Health Research, University of Southern Denmark, 5230 J.B. Winsløws Vej 19, Odense M, Denmark; 3 Research Unit for Applied Health Science, University College South (UC SYD), Lembckesvej 3-7, 6100 Haderslev, Denmark; 4 Section for Biostatistics and Evidence-Based Research, The Parker Institute, Bispebjerg and Frederiksberg Hospital, Nordre Fasanvej 57, 2000 Frederiksberg, Denmark; 5 OPEN – Open Patient data Explorative Network, Odense University Hospital, University of Southern Denmark, J.B. Winsløws Vej 9a, 5000 Odense C, Denmark; 6 Department of Surgery, Vejle Hospital, University Hospital of Southern Denmark, Beriderbakken 4, 7100 Vejle, Denmark; 7 Department of Plastic and Breast Surgery, Aarhus University Hospital, Palle Juul-Jensens, Boulevard 35, 8200 Aarhus N, Denmark; 8 Research Unit of Exercise Epidemiology, Institut for Idræt, University of Southern Denmark, Campusvej 55, 5230 Odense M, Denmark; 9 Research Unit of Rheumatology, Department of Clinical Research, University of Southern Denmark, Odense University Hospital, Campusvej 55, 5230 Odense M, Denmark

**Keywords:** Breast cancer, Late-term shoulder impairments, Rehabilitation, Randomised controlled trial, Study protocol

## Abstract

**Background:**

In breast cancer patients, late-term upper limb sequelae, such as shoulder pain and impaired shoulder function, remain common after primary breast cancer surgery. The aim of this trial is to evaluate whether an expert assessment of shoulder impairments, followed by an individualised treatment plan, is superior to a minimal physiotherapeutic rehabilitation program in reducing shoulder symptoms, among women with late-term shoulder impairments after primary breast cancer.

**Methods/design:**

The study is designed as a stratified, parallel-group, assessor-blinded, randomised, controlled trial conducted in Denmark; 130 participants with late-term shoulder impairments 3–7 years after primary surgery for breast cancer will be recruited. Participants will be randomised (allocation 1:1) to either an expert assessment of shoulder impairments followed by an individualised treatment plan or to follow a minimal physiotherapeutic rehabilitation program delivered in a pamphlet. The primary outcome will be a change in shoulder pain and function from baseline to 12 weeks after initiating the treatment, as measured by the patient-reported outcome Shoulder Pain and Disability Index (SPADI) questionnaire.

**Discussion:**

There has been an insufficient focus in research and clinical practice on late-term shoulder impairment in women following surgery for breast cancer. This trial will focus on interventions towards late-term shoulder impairments and is expected to provide evidence-based knowledge to physiotherapists and women about the management of shoulder pain and impaired shoulder function.

**Trial registration:**

ClinicalTrials.gov NCT05277909. Registered on 11 March 2022.

**Supplementary Information:**

The online version contains supplementary material available at 10.1186/s13063-022-06659-1.

## Background

Breast cancer is the most common cancer in women worldwide [1]. Standard surgical treatment in Denmark is breast-conserving surgery (BCS) or mastectomy in combination with sentinel lymph node dissection (SLND) or axillary lymph node dissection (ALND) [2]. In part due to early diagnosis and optimised treatment methods [3], 5-year survival has improved to currently 87% [4, 5]. Despite fewer mastectomies and more BCS [2, 6], less invasive surgical procedures of the axilla (e.g. fewer ALND vs. SLND) [2, 7, 8], and more refined radiotherapy procedures [3, 9], late-term upper limb impairment still remains common [9, 10]. The most frequent are lymphoedema, sensory disturbances, pain and impaired shoulder function [3, 6, 9–19], with up to 70% of patients reporting at least one of these symptoms three years after surgery [9, 12, 13, 15, 16, 18, 20]. These impairments lead to difficulties in activities of daily living [13, 20], increased risk of depression and anxiety [21, 22] and decreased quality of life (QoL) [21, 23–25].

Previous international research has primarily focused on prevention and treatment of lymphoedema, and less on other upper limb impairments [9, 15, 18, 20, 26, 27]. Preoperative and early postoperative physiotherapeutic interventions are known to be effective in reducing shoulder pain and improving shoulder function after breast cancer treatment [9, 28–30], but there is a lack of international knowledge on the effectiveness of these interventions on the late-term sequelae. The importance of more focus on this is emphasised by studies of a prevalence of up to 50% of impaired shoulder function and pain up to 6 years after surgery [9, 11, 19, 20, 23, 27]. These large numbers might indicate that shoulder pain and impairment is an overlooked long-term health consequence, and a substantial knowledge gap exists as to how to help these women. Currently, no standardised evaluation or treatment of their impairments is offered, and it is therefore up to the individual woman to seek care, resulting in large variations in rehabilitation. Since half of all cases of breast cancer is diagnosed in women aged 62 years or younger [31], i.e. potentially physically active and in the workforce, an improvement in prevention and management of shoulder impairment after breast surgery may substantially benefit both the patients and society. We therefore suggest the present randomised trial whose results may potentially have an immediate impact on clinical practice as well as on long-term outcomes and quality of life after breast cancer surgery.

## Evidence-based research

According to the principles of Evidence-Based Research (EBR) to avoid waste of research, no new studies should be done without a pragmatic review of the existing evidence [32]. We applied the following pragmatic search terms to identify studies; searched on 8 August 2022: {Exercise[tiab] AND shoulder[ti] AND (impairment[tiab] OR pain[tiab]) AND ((“breast cancer” OR breast) AND cancer)}. We found 19 records in database searching, from which 8 records corresponded to randomised trials [28, 33–39]. Thus, the result of the present randomised trial is expected to have a high impact in the health system to in terms of having more focus on late-term shoulder impairments and improving the quality of physiotherapeutic rehabilitation, especially for physiotherapists involved in the management of breast cancer and late-term shoulder impairments.

### Aim and hypothesis

The primary aim of this study is to investigate whether the effect of a patient-centred specialised intervention, consisting of an expert assessment followed by an individualised treatment plan (i.e. *intervention group*), is superior to a minimal physiotherapeutic rehabilitation program delivered in a pamphlet (i.e. *control comparator group)* among women with late-term shoulder impairments 3–-7 years after their primary breast cancer surgery. The hypothesis is that women randomised to the *intervention group* will improve significantly more in shoulder function and pain 12 weeks after initiating the treatment than those randomised to the *control comparator group.*

### Objectives

#### Primary efficacy objective

To compare the effect of the individualised treatment plan, relative to a minimal physiotherapeutic rehabilitation program, on changes in Shoulder Pain and Disability Index (SPADI) from baseline to 12 weeks after initiating the treatment, in women with late-term shoulder impairments after primary breast cancer surgery.

#### Secondary objectives

To compare the effect of the individualised treatment plan, relative to a minimal physiotherapeutic rehabilitation program, after 12 weeks on changes in SPADI pain, SPADI function, SPADI clinical response, impression of the treatments success, active range of motion (A-ROM), passive range of motion (P-ROM), number of treatments received due to shoulder symptoms, maximum shoulder pain intensity, shoulder pain during general activities, shoulder pain at rest, shoulder pain during sleep, and shoulder pain during flexion/rotation/abduction.

#### Exploratory secondary objectives

To compare the effect of the individualised treatment plan, relative to a minimal physiotherapeutic rehabilitation program, after 12 weeks on changes in pain medication consumption, depression score (PHQ-9) and anxiety score (GAD-7).

## Methods/design

### Study design

**Fig. 1:**
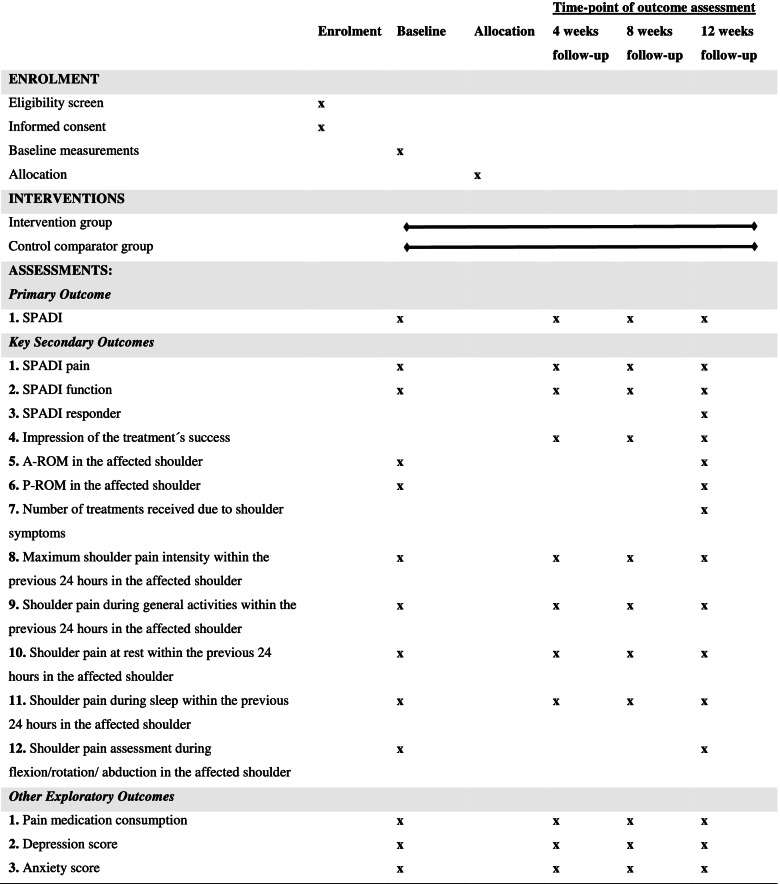
An overview of recommended content for the schedule of enrolment, interventions and assessments

This trial is a stratified (by type of surgery and radiotherapy), block randomised (1:1 allocation), controlled, parallel-group and assessor-blinded superiority trial conducted in Denmark. The primary endpoint for the primary analysis will be 12 weeks after initiating the treatment. Furthermore, 4 and 8 weeks repeated measurements will be included to strengthen the study as supportive evidence. The study protocol follows the “Standard Protocol Items: Recommendations for Interventional Trials (SPIRIT) [40]. See an overview of the recommended content for the schedule of enrolment, interventions and assessments in Fig. 1 and the SPIRIT checklist in Additional file 1. The trial was registered prior to First Patient First Visit at ClinicalTrials.gov (NCT05277909) on 2022–03-11. The recruitment period will begin in April 2022 (i.e. First Patient First Visit) and is expected to be completed in August 2022 (i.e. Last Patient First Visit).

### Setting and locations

The randomised controlled trial is strategically organised at Vejle Hospital, in collaboration between the Department of Physio- and Occupational Therapy, the Department of Surgery and the Department of Orthopaedics. The intervention will be performed in collaboration between the Department of Physio- and Occupational Therapy and the Department of Orthopaedics, Shoulder Sector’s specialised shoulder therapist group.

The Shoulder Sector at Vejle Hospital is a highly specialised unit seeing approximately 2,300 new patients each year. The shoulder sector will be responsible for examining the participant in the *intervention group*.

The Department of Physio- and Occupational Therapy, Vejle Hospital is responsible for recruitment, baseline, randomisation and follow-up examination of both the *intervention group* and *control comparator group*.

### Participants and eligibility criteria

Women who have participated in a parallel performed nationwide cross-sectional questionnaire study (will be published separately) and reported shoulder pain or impairment as their primary discomfort after BCS and mastectomy combined with either SLND or ALND due to breast cancer, and fulfilling the following inclusion and exclusion criteria will be invited to participate in the randomised trial:

#### Inclusion criteria

1. Breast cancer patients who underwent unilateral BCS or mastectomy on the left or right side, including SLND or + / − ALND within the last 3–7 years (2015–2019)
2. Currently living in the Region of Southern Denmark or Central Denmark Region with a radius of 75 km from Vejle Hospital
3. Between 18 and 71 age at the time of surgery for *primary* breast cancer
4. Indicate pain in the chest and/or shoulder area (shoulder impairments) as the biggest problem/late-term effect in everyday life
5. Indicate impaired shoulder function due to pain or due to tightness/tension
6. Indicate shoulder pain at rest, during general activities, during sleep or during flexion, rotation or abduction of the shoulder
7. A score ≥ 15 on the Disabilities of the Arm, Shoulder and Hand (Quick DASH) [41, 42]
8. Agree to participate in this trial and sign written informed consent

#### Exclusion criteria

1. No previous breast cancer (before 2014)
2. Cancer relapse after the date of index surgery, cancer spread outside of thorax and axilla, tumour fixed to the chest wall
3. Primary- or secondary breast reconstruction performed at any time
4. Severe lymphoedema (an average score ≥ 70% in the first 7 questionnaires on the LYMPH-ICF-DK [43, 44]
5. Bilateral breast cancer surgery
6. Previous surgery in the affected shoulder (prior to inclusion)
7. Previous shoulder or upper limb fractures (left/right)
8. Currently receiving chemo-, immuno- or radiotherapy
9. Co-morbidity expected to influence shoulder function (e.g. rheumatoid arthritis, previous stroke, multiple sclerosis)
10. Other reasons for exclusion (e.g. pregnancy, not legally competent, unable to comprehend the information or unable to consent)

### Interventions

#### Intervention group

##### The expert assessment of shoulder impairments and individualised treatment plan

Participants randomised to the *Intervention group* will be referred to an expert assessment of their shoulder impairments at the Shoulder Sector, Vejle Hospital—Orthopaedic Department. The expert assessment will be performed by experienced specialists (e.g. physician and physiotherapist) who are specialised in shoulder diagnostics using x-ray, ultrasound, anamnesis/history and standard clinical tests such as Neer, Hawkin, Jobes Empty Can, Painful Arc and Resisted External Rotation [45–47]. The participant’s diagnosis based on the history, symptoms and clinical findings will be used by the experienced specialist to guide the individualised treatment plan. It is up to the experienced specialist to decide the contents of the treatment plan in each individual case. Typically, it will include referral to physiotherapeutic treatment at the municipality or private practice, or specialised physiotherapeutic rehabilitation at Vejle Hospital. Other treatment options, e.g. ultrasound-guided corticosteroid injection in the shoulder or surgery will be offered at the specialist’s discretion if considered a better choice for the individual woman. Any concomitant interventions are allowed during the trial and will be recorded at the 12-week follow-up, along with the number of visits to any other healthcare professionals (e.g. chiropractor or physiotherapist) at hospital, municipality rehabilitation or private practice due to the shoulder symptoms.

#### Control comparator group

##### A minimal physiotherapeutic rehabilitation program delivered in a pamphlet

Participants randomised to the *control comparator group* will receive a pamphlet from the secretary and be encouraged to perform the exercises at home on a daily basis. This pamphlet contains a program with minimal exercise recommendations for the shoulder consisting of mobility, stretching, strength exercises and tissue treatment. The exercises have been developed in collaboration between specialised physiotherapists from the oncological and orthopaedic Shoulder Sector group at Vejle Hospital. The exercises are assessed particularly suitable for the study target group, who experience late-term shoulder pain and impaired shoulder function after breast cancer surgery.

The purpose of the program is to stimulate circulation, improve shoulder function (mobility), increase muscle strength and reduce shoulder pain. The program consists of three warm-up exercises (arm swing, shoulder rolling and scapula-back pocket exercise) followed by three stretching exercises for the breast and shoulder area. Furthermore, the pamphlet includes a tissue treatment and four strength exercises for the shoulder (external rotation, extension and flexion of the shoulder and diagonal pull apart). Mobility (with 5–10 repetitions), stretching exercises (in 30 s) and tissue treatments will be performed twice a day, while the strength exercises will be performed once a day with 3 × 12 repetitions. The participants will be encouraged in writing to work to their pain threshold to improve shoulder function and reduce shoulder pain.

Also, in the control comparator group, any concomitant interventions will be allowed during the trial, while the number of visits to a healthcare professional will be collected at 12-week follow-up.

### Recruitment procedure

As the background population of potentially eligible participants is not routinely followed-up with standard evaluations of their potential late-term adverse events, participants will be recruited through a letter inquiry, based on a register extraction, including an invitation to participate in a cross-sectional survey of late-term adverse events.

More specifically, a register extraction from the Danish Health Data Authority of women between 18 and 71 years of age at the time of primary breast cancer surgery, and who underwent BCS or mastectomy on the left or right side, including SLND or ALND in 2015–2019, will be performed. The letter inquiry will invite the women to participate in the cross-sectional survey, by clicking actively on a link to participate. The link will lead to the survey hosted on a secure server by Odense Patient data Explorative Network (OPEN), data will be stored in a Research Electronic Data Capture (REDCap) database [48].

Women will automatically be assessed for initial eligibility, based upon their answers in the survey, and, if deemed eligible, provided with further information about the trial and the possibility for giving informed consent (by actively ticking a box in the survey) to be contacted by phone by a project coordinator for further information and possible recruitment to the randomised controlled trial. Eligible women will be contacted based upon a randomised sequence, to counteract any possible bias in relation to the order in which the women have answered the survey. If women are interested in participating, written information about the trial, e.g. the design with allocation to either of the two interventions, will be sent electronically. The women will be recommended taking at least 24 h to consider participation with a relative.

Within 24–72 h, a secretary at the Department of Physio- and Occupational Therapy will contact the women, who gave informed consent to be contacted by phone, and ask if they wish to participate in the study. An appointment for the baseline assessment is made with the women still interested in participating. The project coordinator and secretary involved in the recruitment will be trained and instructed in the recruitment procedure to secure an unbiased recruitment.

On the day for baseline measurements, the secretary at the Department of Physio- and Occupational Therapy will obtain written informed consent prior to baseline measurements.

Immediately after obtaining the written informed consent, an outcome assessor performs the clinical baseline assessment. The outcome assessors who performed the clinical baseline assessment will also perform the 12-week follow-up, blinded towards treatment allocation.

**Fig. 2:**
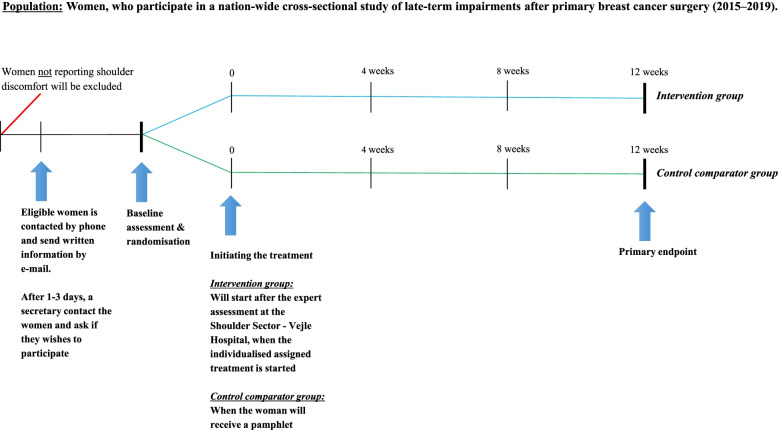
Overview of the recruitment procedure. Intervention group: expert assessment of shoulder impairments and individualised treatment plan. Control comparator group: minimal physiotherapeutic rehabilitation program delivered in a pamphlet. SPADI, Shoulder Pain and Disability Index

After baseline assessment, the secretary will with a REDCap randomise system randomise the participants, to either the *intervention group* or *control comparator group* intervention. The randomisation is performed either on the day where the result is revealed immediately, or the participant is contacted by telephone 1–2 days after the baseline assessment. If randomised to the *intervention group*, the secretary will refer the participant for examination in the Shoulder Sector. If randomised to the *control comparator group*, the secretary will hand out the exercise pamphlet. Secondly, the secretary will book an appointment for follow-up measurements for all participants, respectively 12 weeks after participants’ first visit in the shoulder sector (*Intervention group*) or after delivering the exercise pamphlet (*control comparator group*). A schematic overview of the recruitment procedure and milestones in the trial is illustrated in Fig. 2.

#### Randomisation and allocation concealment

Eligible participants will randomly be assigned in permuted blocks of 2 to 6, with a 1:1 allocation, based on a computer-generated randomisation list generated by an independent data manager implemented into the REDCap randomised system, to either the *intervention group* or *control comparator group.* Participants will be stratified in 5 groups according to the type of surgery and + / − radiotherapy treatment ((1) BCS and SLND + radiotherapy, (2) BCS and SLND − radiotherapy, (3) BCS and ALND + radiotherapy, (4) mastectomy and SLND − radiotherapy and (5) Mastectomy and ALND + radiotherapy). To ensure concealment, the primary investigator, assessors and administrators of the randomisation will be blinded to the block sizes, as the randomisation code will be stored in REDCap, with no access for the project group.

#### Blinding

Outcome assessors who perform baseline and follow-up will be blinded to group allocation. At baseline the assessment is performed prior to randomisation. To ensure blinding of the outcome assessors at follow-up, participants will be encouraged not to reveal their allocated intervention. An independent biostatistician, blinded to specific group allocation, will supervise the statistical analyses. Due to the study design, the participants, orthopaedic specialists, secretary and physiotherapists involved in the interventions cannot be blinded to treatment allocation. However, none of these will participate in the data analysis or preparation of the manuscript.

### Crossover and withdrawal

To reduce crossover and withdrawal, preventive initiatives will be taken. The secretary involved in randomisation and delivering the pamphlet to the *control comparator group* will be trained to encourage participants to continue the exercises for minimum of 12 weeks. One week prior to 12-week follow-up, a secretary will contact the participants in the *Control comparator group* to ensure that they remember their 12-week follow-up appointment and reschedule if participants are unable to attend. In general, the risks of crossover in a trial like this with a short intervention period on three months are anticipated to be low. However, to reduce the risk of crossover from *control comparator group* to the *intervention group*, participants randomised to the *control comparator group* will be informed that they will be able to be referred for an individual examination at the Shoulder Sector after the 12-week follow-up, if their pain and function has not improved clinically relevant.

Participants from the *intervention group* experiencing worsening of their symptoms and contacting the primary investigator or the Orthopaedic Department will be assessed by an orthopaedic specialist, who performed the initial assessments during the recruitment phase. If participants from the *control comparator group* are seen for an assessment at the Orthopaedic Department, they will be registered as a crossover, regardless of further treatment.

The reason for each crossover or withdrawal will be registered. Participants performing a crossover will remain in the study and be analysed in the intention-to-treat analysis as randomised.

### Outcome measures

Participant background demographic variables median age, height, weight, body mass index (BMI), co-morbidities, depression, anxiety, pain medication consumption due to shoulder-related pain, index shoulder, dominant side affected, shoulder symptom and duration will be collected at baseline.

#### Primary outcome

- 1: Change in Shoulder Pain and Disability Index (SPADI) from baseline to 12 weeks after initiating the treatment.

SPADI is a 13-item patient-reported outcome measure to assess shoulder pain (5 items) and shoulder function (8 items) within the last week. The items are scored on a numeric rating scale that ranges from 0 (no pain/no difficulty) to 10 (worst pain/so difficult that required help) [41, 49]. Each domain score is equally weighted and added to a total percentage score that ranges from 0 (best) to 100 (worst). The higher the score, the greater the patient-reported shoulder impairments. This region-specific questionnaire can be used in patients with various or unspecified shoulder diagnoses [49]. SPADI is a valid, reliable and responsible measure among patients with shoulder impairments [41, 49] [time frame: 0, 4, 8 and 12 weeks].

#### Key secondary outcomes

Key secondary outcome measures will include:

- 1: Change in SPADI pain from baseline to 12 weeks after initiating the treatment.
  Change in SPADI pain will be reported as a separate subscale [50]. The 5-item pain subscale is scored on a numeric rating scale that ranges from 0 (no pain) to 10 (worst pain) [41, 49]. The higher the score, the greater the patient-reported shoulder pain and reduction in the SPADI pain score will suggest improvement [time frame: 0, 4, 8 and 12 weeks].
- 2: Change in SPADI function from baseline to 12 weeks after initiating the treatment.
  Change in SPADI function will be reported as a separate subscale [50]. A 6-item version (exclusion of questions three and seven) of the disability subscale exhibited adequate fit in the Danish version [50]. The 6-item disability subscale is scored on a numeric rating scale that ranges from 0 (no difficulty) to 10 (so difficult that required help) [41, 49]. The higher the score, the greater the patient-reported shoulder disabilities and reduction in the SPADI function score will suggest an improvement [time frame: 0, 4, 8 and 12 weeks].
- 3: SPADI clinical response.
  Response to treatment will be computed for the SPADI change score for each woman in both treatment groups and presented dichotomised into responder and non-responder as number and percentages of responders. Women will be classified as responders if the SPADI change score improves by 8 points or more (≥), corresponding to the minimal clinically important difference on SPADI [51, 52] from baseline to 12 weeks follow-up [time frame: 12 weeks (follow-up)].
- 4: Global perceived effect (GPE) measured at 4, 8 and 12 weeks after initiating the treatment.
  The GPE will evaluate the impression of the treatment’s success including overall shoulder problems on a 7-point Likert scale ranging from “markedly worse” to “markedly improved” [53, 54] [time frame: 4, 8 and 12 weeks].
- 5: Change in active range of motion (A-ROM) in the affected shoulder from baseline to 12 weeks after initiating the treatment.
  A smartphone inclinometer (GetMyROM) [55–59] will be used to assess A-ROM in flexion, internal rotation, external rotation and abduction respectively on the operated side. After one test trial, the mean value of three measurements will be taken for both flexion, rotation and abduction respectively on the operated side [time frame: 0 and 12 weeks].
- 6: Change in passive range of motion (P-ROM) in the affected shoulder from baseline to 12 weeks after initiating the treatment.
  A smartphone inclinometer (GetMyROM) [55–59] will be used to assess P-ROM in flexion, internal rotation, external rotation and abduction respectively on the operated side. After one test trial, the mean value of three measurements will be taken for both flexion, rotation and abduction respectively on the operated side. [Time Frame: 0 and 12 weeks].
- 7: Number of treatments received for shoulder symptoms from baseline to 12 weeks after initiating the treatment.
  Number of visits to a healthcare professional (e.g. physician, chiropractor or physiotherapist) at hospital, municipality rehabilitation or private practice due to the shoulder symptoms during the intervention period, will be collected by using a patient-reported questionnaire [time frame: 12 weeks (follow-up)].
- 8: Change in maximum shoulder pain intensity within the previous 24 h in the affected shoulder measured by the Numeric Rating Scale (NRS) from baseline to 12 weeks after initiating the treatment.
  The NRS pain scale is a single 11-item patient-reported outcome measure used to assess the maximum shoulder pain intensity. The scale ranges from 0 (no pain) to 10 (worst pain imaginable). The higher the score, the greater the patient-reported shoulder pain intensity and a reduction in the NRS score will suggest an improvement. The NRS is a reliable, valid and responsive measure of pain in patients with cancer [60] [time frame: 0, 4, 8 and 12 weeks].
- 9: Change in shoulder pain during general activities within the previous 24 h in the affected shoulder measured by the Numeric Rating Scale (NRS) from baseline to 12 weeks after initiating the treatment.
  The NRS pain scale is a single 11-item patient-reported outcome measure used to assess pain during general activities. The scale ranges from 0 (no pain) to 10 (worst pain imaginable). The higher the score, the greater the patient-reported shoulder pain and a reduction in the NRS score will suggest an improvement. The NRS is a reliable, valid and responsive measure of pain in patients with cancer [60] [time frame: 0, 4, 8 and 12 weeks].
- 10: Change in shoulder pain at rest within the previous 24 h in the affected shoulder measured by the Numeric Rating Scale (NRS) from baseline to 12 weeks after initiating the treatment.
  The NRS pain scale is a single 11-item patient-reported outcome measure used to assess pain at rest. The scale ranges from 0 (no pain) to 10 (worst pain imaginable). The higher the score, the greater the patient-reported shoulder pain and a reduction in the NRS score will suggest an improvement. The NRS is a reliable, valid and responsive measure of pain in patients with cancer [60] [time frame: 0, 4, 8 and 12 weeks].
- 11: Change in shoulder pain during sleep within the previous 24 h in the affected shoulder measured by the Numeric Rating Scale (NRS) from baseline to 12 weeks after initiating the treatment.
  The NRS pain scale is a single 11-item patient-reported outcome measure used to assess pain during sleep. The scale ranges from 0 (no pain) to 10 (worst pain imaginable). The higher the score, the greater the patient-reported shoulder pain and a reduction in the NRS score will suggest an improvement. The NRS is a reliable, valid and responsive measure of pain in patients with cancer [60]. [Time Frame: 0, 4, 8 and 12 weeks].
- 12: Change in shoulder pain during flexion/rotation/abduction in the affected shoulder measured by Numeric Rating Scale (NRS) from baseline to 12 weeks after initiating the treatment.

The NRS pain scale is a single 11-item patient-reported outcome measure used to assess pain during flexion/rotation/abduction. The scale ranges from 0 (no pain) to 10 (worst pain imaginable). The higher the score, the greater the patient-reported shoulder pain and a reduction in the NRS score will suggest an improvement. The NRS is a reliable, valid and responsive measure of pain in patients with cancer [60] [time frame: 0 and 12 weeks].

#### Other exploratory outcomes

The other exploratory outcomes are measured at baseline and will include:

- 1: Change in pain medication consumption from baseline to 12 weeks after initiating the treatment.
  Pain medication consumption in the past week due to shoulder-related pain including questions about yes/no, type (prescription or non-prescription medicine) and frequency will be collected by using a patient-reported questionnaire [time frame: 0, 4, 8 and 12 weeks].
- 2: Change in Patient Health Questionnaire – 9 (PHQ-9) from baseline to 12 weeks after initiating the treatment.
  PHQ-9 is a 9-item patient-reported outcome measure to assess depression within the last 2 weeks. The total score ranges from 0 to 27; 5–9 = minimal symptoms, 10–14 = minor/mild depression, 15–19 = major depression, moderately severe, >20 = major depression, severe [61, 62]. The higher the score, the greater the patient-reported severe depression and reduction in the PHQ-9 score will suggest improvement. PHQ-9 is a reliable and valid measure of depression [62–65] in cancer patients and the general population [61] and can measure changes over time [62, 63] [time frame: 0, 4, 8 and 12 weeks].
- 3: Change in General Anxiety Disorder – 7 (GAD-7) from baseline to 12 weeks after initiating the treatment.

GAD-7 is a 7-item patient-reported outcome measure to assess anxiety within the last 2 weeks. The total score ranges from 0 to 21; 0–4 = minimal anxiety symptoms, 5–9 = mild anxiety symptoms, 10–14 = moderate anxiety symptoms, >15 severe levels of anxiety symptoms [62, 66, 67]. The higher the score, the greater the patient-reported severe anxiety and a reduction in the GAD-7 score will suggest an improvement. GAD-7 is a reliable and valid measure of anxiety in cancer patients and the general population [66, 67] [time frame: 0, 4, 8 and 12 weeks].

Table 1 presents the data to be collected.

**Table 1:** Presents all outcome variables, data collection instruments, measures, time points and statistical analysis

**Outcome variables**	**Data collection instrument**	**Measures**	**Time points of outcome assessment**	**Statistical analysis**
**Primary outcome**				
**1.** Change in shoulder pain and function	SPADI (questionnaire)	Score 0–100 (continuous)	0, 4, 8 and 12 weeks	Repeated-Measures, Mixed Effects Model
**Key secondary outcomes**				
**1.** Change in shoulder pain	SPADI (questionnaire)	Score 0–10 (continuous)	0, 4, 8 and 12 weeks	Repeated-Measures, Mixed Effects Model
**2.** Change in shoulder function	SPADI (questionnaire)	Score 0–10 (continuous)	0, 4, 8 and 12 weeks	Repeated-Measures, Mixed Effects Model
**3.** Clinical response	SPADI minimal important change criteria^a^—no. (%)	SPADI change score (dichotomous)	12 weeks (follow-up)	Logistic regression after 12 weeks
**4.** Impression of the treatment´s success	GPE (questionnaire)	Score 0–7 (continuous)	4, 8 and 12 weeks	Repeated-Measures, Mixed Effects Model
**5. and 6.** Change in A- and P-ROM in the affected shoulder	Smartphone Inclinometer test protocol	Degree (continuous)	0 and 12 weeks	ANCOVA
**7.** Number of treatments received due to shoulder symptoms^b^	Questionnaire	Mean number of received treatments (continuous)	12 weeks (follow-up)	ANCOVA
**8.** Change in maximum shoulder pain intensity within the previous 24 h in the affected shoulder	NRS in questionnaire	Score 0–10 (continuous)	0, 4, 8 and 12 weeks	Repeated-Measures, Mixed Effects Model
**9.** Change in shoulder pain during general activities within the previous 24 h in the affected shoulder	NRS in questionnaire	Score 0–10 (continuous)	0, 4, 8 and 12 weeks	Repeated-Measures, Mixed Effects Model
**10.** Change in shoulder pain at rest within the previous 24 h in the affected shoulder	NRS in questionnaire	Score 0–10 (continuous)	0, 4, 8 and 12 weeks	Repeated-Measures, Mixed Effects Model
**11.** Change in shoulder pain during sleep within the previous 24 h in the affected shoulder	NRS in questionnaire	Score 0–10 (continuous)	0, 4, 8 and 12 weeks	Repeated-Measures, Mixed Effects Model
**12.** Change in shoulder pain during flexion/rotation/abduction in the affected shoulder	NRS / test protocol	Score 0–10 (continuous)	0 and 12 weeks	ANCOVA
**Other exploratory outcomes**				
**1.** Pain medication consumption	Questionnaire	Type (categorical)	0, 4, 8 and 12 weeks	Logistic regression
**2.** Depression score	PHQ-9 (questionnaire)	Score 0–27 (continuous)	0, 4, 8 and 12 weeks	ANCOVA
**3.** Anxiety score	GAD-7 (questionnaire)	Score 0–21(continuous)	0, 4, 8 and 12 weeks	ANCOVA
**Sensitivity analyses**				
Per-protocol analysis^c^				ANCOVA
As-treated analysis^**c**^				ANCOVA

^a^Patients will be classified as having a clinically relevant change if the SPADI score improves by 8 points or more [51, 52]

^b^Number of visits to healthcare professionals (e.g. physician, chiropractor or physiotherapist) at hospital, municipality rehabilitation or private practice due to the shoulder symptoms during the intervention period

^c^Primary outcome. SPADI Shoulder Pain and Disability Index, GPE global perceived effect, NRS Numeric Pain Intensity Rating Scale, PHQ-9 Patient Health Questionnaire—9, GAD-7 General Anxiety Disorder – 7

### Data collection

Baseline and follow-up assessments will be performed by blinded outcome assessors. Before starting the data collection, the primary investigator will introduce the assessors to the test manual and subsequently decide a standard interpretation of all outcome variables. Baseline characteristics and patient-reported outcomes (baseline and follow-up) will be collected by using online questionnaires. At baseline and 12-week follow-up, women will answer the patient-reported outcomes in an undisturbed examination room at Vejle Hospital. At 4- and 8-week follow-up, an e-mail including a link to the online questionnaires will be sent to the participants. If a woman does not reply within three days, a reminder e-mail will be sent. The participant will be contacted by telephone within 4 days after the reminder is sent out, in case of still no reply.

### Data management

All data collected in this trial will be treated, managed and stored strictly confidentially in REDCap, OPEN [48]. To ensure no missing items from the patient-reported outcome questionnaires, the “Required fields” option will be activated. Data quality is ensured through answer validity and double data entry, when entered in the functional performance data in REDCap by outcome assessors. Each participant is labelled with an ID-number in the REDCap-database to ensure pseudo-anonymity. Personal data will be kept separate from the main data to protect confidentiality throughout each phase of this trial. To analyse data all electronic data will be uploaded encrypted to a password-secured server (Region of Southern Denmark) to comply with current data protection standards. The raw data set will be stored for five years after completing the trial, while the anonymised dataset will be available for the corresponding author on reasonable request. After the publication of this trial an anonymised patient-level dataset and corresponding statistical code will be made publicly available if required by the scientific journal, in which the results are published.

#### Data monitoring and registration of SAE

Serious adverse events (SAE) are anticipated to be rare, and no formal data monitoring committee will be used. SAE will be collected from the medical record review conducted at the 12-week follow-up. Furthermore, a short patient-reported questionnaire at the 12-week follow-up will be used to ensure that all SAE are recorded.

In practice when adverse events occur, physiotherapists or participants contact the primary investigator who makes a note in the medical records. The trial management committee (i.e. the author group) will discuss any serious adverse events or outcomes occurring during baseline to 12-week follow-up (primary outcome). Subsequently, the primary investigator will report these to the ethics committee and monitor the number of SAEs. SAEs will be categorised in accordance with the definitions established by the United States Food and Drug Administration (e.g. hospitalisation or death) [68].

#### Sample size and power considerations

In the period 2015–2019, 4300 breast cancer surgeries were performed in the Region of Southern Denmark [69–71]. A 40% participation rate is expected to reply on a cross-sectional survey [72] and will match the general inclusion criteria regarding unilateral surgery and no previous history of breast cancer. 1/3 of these women are expected to have upper limb impairments such as shoulder pain and impaired shoulder function [12, 19, 23] and 1/3 are expected to decline the offer to participate, resulting in an eligible population of approximately *n* = 384 women.

In order to achieve a priori statistical power of at least 85%, with a two-sided significance level *α* = 0.05, with an anticipated standard deviation (SD) = 15.41 SPADI units [73], the estimated total sample size is *n* = 130 (~ 65 participants in each group), to be able to detect a minimal clinically relevant difference defined as 8 points on the SPADI-score [51, 52].

#### Statistical methods

The Consolidating Standards of Reporting Trials (CONSORT) guideline [74] will be followed in all trial reporting aspects, as recommended by the *“Enhancing the QUAlity and Transparency Of health Research”* (EQUATOR) network website [75]. The primary analyses will be based on the Intention to Treat (ITT) population, i.e. based on the Full Analysis Set (having the outcome of interest measured at baseline). The ITT principle asserts the effect of a treatment policy (that is, the planned treatment regimen), rather than the actual treatment given (i.e. it is independent of treatment adherence). Accordingly, participants allocated to a treatment group (*X*_I_ and *X*_C_, respectively) will be followed up, assessed and analysed as members of that group, irrespective of their adherence to the planned course of treatment (i.e. independent of withdrawals and cross-over phenomena)[76]. All 95% confidence intervals and *P-*values will be two-sided. Explicit adjustments for multiplicity will not be applied, but rather an analysis of the confirmatory secondary outcomes in a prioritised order: The analyses of the key secondary outcomes will be performed in sequence until one of the analyses fails to show the statistically significant difference, or until all analyses have been completed at a statistical significance level of α = 0.05 [77]. The key secondary statistical tests will be reported with P values for hypothesis tests and claims of statistical significance.

The primary (continuous) outcome will be analysed by using repeated measures mixed linear models, including participants as a random effect, with fixed factors for group (2 levels) and week (4 levels for the SPADI questionnaire [weeks 0, 4, 8, and 12]) and the corresponding interactions, adjusted for baseline values. To assess the adequacy of the linear models describing the observed data—and checking assumptions for the systematic and the random parts of the models—the model features will be investigated via the predicted values and the residuals; that is, the residuals have to be normally distributed (around 0) and be independent of the predicted values. Results will be expressed based on least squares mean estimates as well as the differences in the changes from baseline, with 95% CIs to represent the precision of the estimates. Further, for the primary outcome, a 95% CI excluding differences greater than 4 SPADI points between groups will be interpreted as indicating the absence of a clinically meaningful difference. Dichotomous outcome variables will be analysed with logistic regression, with identical fixed effect factors and covariates as the mixed linear model described above. Missing data for dichotomous outcomes will be computed based on conservative (non-responder) imputations.

To handle missing data, repeated-measures linear mixed models will be used [77–81]. The following four strategies for interpretation of missing data will be applied in the ITT analysis: “1. Attempt to follow up all randomised participants, even if they withdraw from allocated treatment, 2. Perform a main analysis of all observed data that are valid under a plausible assumption about the missing data, 3. Perform sensitivity analyses to explore the effect of departures from the assumption made in the main analysis, and 4. Account for all randomised participants, at least in the sensitivity analyses” [79].

Sensitivity analyses will be performed on various population analyses, including a non-responder imputation, per-protocol and as-treated analysis, to examine the robustness by revealed similar results in these sensitivity analyses.

Subgroup analyses [82] will be used to examine whether the observed overall treatment effect varies across participants’ subgroups, and to whether the effect is modified by the value of a variable assessed at baseline: analysed by thresholds median age, median duration of shoulder symptoms, obesity (BMI ≥ 30 kg/m^2^), dominant side affected (left vs right). This statistical approach to evaluate potential effect modifiers will be a test for statistical interaction on whether the treatment effect (net benefit SPADI score) varies across levels of the effect modifier [83].

All data analysis will be performed applying STATA 17 and SAS software.

#### Interim analysis and early stopping rule

Patient recruitment stops when a total number of 130 patients have been included and finished their intervention or when the deadline of 31 August 2022 is reached.

## Ethics and dissemination

The primary ethical considerations of the current trial will be, as consequence of the design of the trial, deprival of the *intervention group* for 65 women allocated to receive the *control comparator group* intervention. This may be justified due to several reasons. Firstly, no standardised treatment procedure exists for late-term shoulder pain and function 3–7 years after primary surgery for breast cancer, and it is therefore uncertain how to help these women. Furthermore, no direct comparison between the *intervention* and *control comparator group* has been conducted, and thus it is unknown whether the *intervention group* is superior to the *control comparator group* intervention. Secondly, both intervention strategies are expected to reduce shoulder pain and improve shoulder function. Thirdly, patients randomised to the *control comparator group* will have the possibility for a referral to an individual intervention after the trial period.

### Adverse events or harms

In general, the risks of serious adverse events or harms, e.g. musculoskeletal injuries from participating in this trial are anticipated to be low. The ultrasound and orthopaedic clinical tests used in this trial have no documented risks or harms. The X-ray used includes a risk associated with the radiation itself and the size of the radiation dose. Since the participants are only x-rayed once during this trial and the radiation dose is 0.2 mSV (milliSievert) per examination, the risk of developing cancer due to this radiation is 1 in 1,000,000 (corresponding to 0.001%). These values are therefore considered to be at lower risk (category IIa), and the utility assessed higher by the fact that this trial can be expected to provide increased knowledge and health benefits.

## Discussion

The main limitation of this trial is that the participants, orthopaedic specialists, secretary and physiotherapists involved in the interventions cannot be blinded to treatment allocation. In addition, it may be argued that some of the women included in the trial may have other explanations for their shoulder impairment than their previous breast cancer surgery. For that reason, we deliberately include only women who have developed shoulder pain after their primary breast cancer surgery and exclude women with co-morbidities expected to influence shoulder function (e.g. rheumatoid arthritis, multiple sclerosis) or have had previous shoulder surgery or previous fractures in the shoulder or upper limb. Nevertheless, this trial we only can comment on whether the interventions work on the actual shoulder pain and function in women who have been treated for primary breast cancer. A strength of this trial is that it is an assessor-blinded, randomised controlled design that include a blinded analysis and interpretation with a priori registration protocol ensuring transparency.

This trial will focus on evaluating if a well-established standard approach to patients referred to hospital with shoulder pain also can benefit women with late-term shoulder impairments after breast cancer treatment. Many breast cancer survivors accept having pain as a consequence of their primary treatment, and currently, many of them do not receive any intervention unless they actively seek help. The intervention will be evaluated in a methodologically strong study and will provide evidence-based knowledge to healthcare professionals and patients about the management of shoulder pain and impaired shoulder function among these women affected by late-term sequelae.

## Trial status

The third version of the ethical protocol was accepted on 15 February 2022. At the time of submission of this study protocol, the trial will start to recruit women. The recruitment starts in April 2022 and is expected to end in August 2022.

## Supplementary Information

**Additional file 1.**

**Additional file 2.**

## Data Availability

The datasets generated and analysed during the current study are available from the corresponding author on reasonable request.

## Abbreviations

BCS: Breast-conserving surgery
SLND: Sentinel lymph node dissection
ALND: Axillary lymph node dissection
QoL: Quality of life
Quick DASH: Disabilities of the Arm, Shoulder and Hand
SPADI: Shoulder Pain and Disability Index
NRS: Numeric Pain Intensity Rating Scale
PHQ-9: Patient Health Questionnaire
GAD-7: General Anxiety Disorder
GPE: Global perceived effect
OPEN: Odense Patient Data Explorative Network
REDCap: Research Electronic Data Capture
ITT: Intention-to-treat
ANCOVA: ANalysis of COVAriance
SD: Standard deviation
EQUATOR: Enhancing the QUAlity and Transparency Of health Research network
CONSORT: CONsolidating Standards Of Reporting Trials
