# Supplementary material for: Effectiveness of an expert assessment and individualised treatment compared with a minimal home-based exercise program in women with late-term shoulder impairments after primary breast cancer surgery: study protocol for a randomised controlled trial
Source: Trials. 2022 Aug 20;23:701. doi: 10.1186/s13063-022-06659-1 (PMC9392220; doi:10.1186/s13063-022-06659-1)
Supplement: Supplementary file 2 — Additional file 2. [file 13063_2022_6659_MOESM2_ESM.pdf]

Informeret samtykke til deltagelse i et sundhedsvidenskabeligt forskningsprojekt

Forskningsprojektets titel:
"Specifik eller generel behandling af vedvarende senfølger i skulderregionen efter brystkræftoperation – Hvad virker bedst?" (Lodtrækningsforsøg)

Erklæring fra forsøgspersonen:
Jeg har fået skriftlig og mundtlig information, og jeg ved nok om formål, metode, fordele og ulemper til at sige ja til at deltage.

Jeg ved, at det er frivilligt at deltage, og at jeg altid kan trække mit samtykke tilbage uden at miste mine nuværende eller fremtidige rettigheder til behandling.

Jeg giver samtykke til at deltage i forskningsprojektet, og til at forsøgsansvarlige, sponsor og sponsors repræsentanter samt kontrolmyndighed direkte må tilgå min patient- og sundhedsjournal med henblik på indhentning af de i det skriftlige materiale angivne informationer (jf. dokument *"Deltagerinformationsmateriale vedrørende forskningsprojektet"*), og har fået en kopi af dette samtykkeark samt en skriftlige information om projektet til eget brug.

Forsøgspersonens navn: ____

Dato: ________________ Underskrift:____

Ønsker du at blive informeret om forskningsprojektets resultat?:

Ja______(sæt x) Nej______(sæt x)

Erklæring fra den, der afgiver information:
Jeg erklærer, at forsøgspersonen har modtaget mundtlig og skriftlig information om forsøget.

Efter min overbevisning er der givet tilstrækkelig information til, at der kan træffes beslutning om deltagelse i forsøget.

Navnet på den, der afgiver information:

Dato: _____________ Underskrift: ______________________________________

Projektidentifikation (projekt-ID): S-20200021
